# Poorly controlled ambulatory blood pressure in outpatients with peripheral arterial disease

**DOI:** 10.48101/ujms.v126.7609

**Published:** 2021-04-29

**Authors:** Nina Dahle, Emma Skau, Jerzy Leppert, Johan Ärnlöv, Pär Hedberg

**Affiliations:** a Centre for Clinical Research, Uppsala University, Falun, Sweden; b Primary Health Care Center Britsarvet-Grycksbo, County of Dalarna, Falun, Sweden; c Centre for Clinical Research, Uppsala University, Västmanland County Hospital, Västerås, Sweden; d Department of Cardiology, Danderyd University Hospital, Stockholm, Sweden; e Division of Family Medicine and Primary Care, Department of Neurobiology, Care Sciences and Society, Karolinska Institutet, Huddinge, Sweden; f School of Health and Social Studies, Dalarna University, Falun, Sweden; g Department of Clinical Physiology, Västmanland County Hospital, Västerås, Sweden

**Keywords:** Carotid artery disease, cardiovascular risk factors, hypertension, smoking, hyperlipidemia, preventive efforts

## Abstract

**Background:**

Patients with peripheral arterial disease (PAD) are generally less intensively managed than patients with coronary heart disease (CHD), despite that their risk of complications is believed to be equivalent. Identification of PAD patients at risk of poorly controlled blood pressure (BP) could lead to improved treatment, thus lowering the risk of cardiovascular (CV) complications. We aimed to describe the prevalence of poorly controlled cardiovascular (CV) risk factors, focusing on BP, in outpatients with PAD diagnosed in a vascular ultrasound laboratory.

**Methods:**

Consecutive outpatients with carotid and/or lower extremity PAD were included (*n =* 402) and examined with blood sampling, clinical BP, and 24-h ambulatory BP measurements. A poorly controlled clinical BP was defined as ≥140/90 mmHg, ambulatory BP ≥130/80 mmHg, low-density lipoprotein (LDL)-cholesterol level ≥2.5 mmol/L, and glycated hemoglobin (HbA1c) level >53 mmol/mol in those with diabetes.

**Results:**

Most of the patients had poorly controlled clinical (76.6%) and ambulatory BP (51.7%) profiles. Antihypertensive medications were prescribed in 84% of the patients. However, >40% of them used only 0–1 medication, and <25% of them used three or more agents. Clinical BP, a low number of medications, body mass index, and the presence of diabetes independently predicted a poorly controlled ambulatory BP. Nearly one-third of the patients were smokers, and most of the cohort had an LDL-cholesterol level of ≥2.5 mmol/L. An HbA1c level of >53 mmol/mol was present in 55% of diabetic patients.

**Conclusion:**

Poorly controlled clinical and ambulatory systolic BP profiles were common. In addition, suboptimal control of other important CV risk factors was detected. The findings of this study highlight the need for better preventive efforts against CV risk factors in outpatients with PAD.

## Introduction

Peripheral arterial disease (PAD) is a common clinical manifestation of systemic arterial atherosclerosis, affecting arteries other than the coronary arteries, intracranial arteries, and the aorta (1, 2). Atherosclerosis is often generalized, and many affected patients also have coronary artery disease (CAD) (2). Patients can present with a spectrum of symptoms, such as claudication, but most are asymptomatic, and all face a substantially increased risk of major cardiovascular (CV) events and deaths (3, 4). PAD is the third leading cause of CV morbidity, after CAD and stroke (5). It is considered to be equivalent to the coronary heart disease risk, warranting aggressive secondary prevention against CV risk factors. However, patients with PAD are generally less intensively managed compared with those with CAD (1, 6–9).

The overall prevalence of hypertension in adults is about 30–45%, and it becomes more common with advancing age (10). Among registered diagnoses in hospital care and primary health care in Sweden, hypertension is the diagnosis with the highest prevalence (11, 12). Antihypertensive treatments are well established, safe, and highly effective (13). However, many patients with hypertension have inadequate control of their BP or undergo no treatment at all (10). Some of the factors found to be associated with poorly controlled BP in the general population include diabetes, older age, obesity, multi-drug regimens, lack of information on hypertension, and living alone (14–18). There is growing evidence that ambulatory BP measurement (ABPM) is a strong predictor of organ damage and CV outcomes. It can provide important clinical information beyond clinical BP measurements, such as revealing nocturnal dipping patterns (19–25). However, how ambulatory BP is controlled and how ABPM can be useful in patients with PAD is not well studied. To further identify patients with PAD at risk of poor BP control could lead to individualized and improved BP treatments, and thereby a lower risk for CV complications (15). In this study, we aimed to describe the prevalence of poorly controlled CV risk factors, focusing on ambulatory BP in consecutive outpatients with PAD diagnosed in a vascular ultrasound laboratory.

## Methods

### Study population

Analyses were based on patients included in the Peripheral Arterial Disease in Västmanland (PADVa) study (26). All patients visiting the ultrasound laboratory of the Department of Vascular Surgery at the Västmanland County Hospital in Västerås, Sweden, from April 2006 to February 2011, were considered for inclusion. Reasons for referral include claudication (45%), transient ischemic attack or stroke (26%), aortic aneurysm (8%), heart murmur (5%), suspected renal artery stenosis or renovascular hypertension (4%), and others (12%). Every patient was examined with ultrasonography to identify any stenosis in the internal carotid artery (ICA). Patients with symptoms of claudication also underwent ankle BP measurement to calculate the ankle–brachial index (ABI) and ultrasonography of the arteries in the symptomatic leg. The patients were invited to participate in the PADVa study if they met at least one of the following inclusion criteria: 1) mild to severe stenosis or occlusion of the ICA, 2) symptoms of claudication combined with ABI ≤0.90 in the symptomatic lower extremity, or 3) symptoms of claudication combined with ultrasonographic evidence of arterial occlusive disease in the same extremity.

In total, 452 patients (73.6%) accepted the invitation to join the study. Everyone in the study was offered ABPM, of whom 35 individuals refused. We excluded patients with <10 daytime or <5 night-time ABPM readings (*n =* 15) (27), leaving 402 patients for analysis.

The study was approved by the Ethics Committee of Uppsala University, Sweden (Dnr 2005:382). All participants gave their written informed consent to participate. The study is registered with ClinicalTrials.gov number NCT01452165.

### Examination protocol

All patients were invited to the Department of Clinical Physiology and were examined according to a standard examination protocol, comprising a questionnaire, including the number of persons in household (living alone vs. cohabitating), educational level (low level was defined as primary school or less), smoking status (smoking defined as regular smoking within the past year), medical history, medication, and physical activity (physically inactive was defined as mostly sedentary with more demanding activities, such as walking, biking, gardening < 2 h per week). Self-reported diagnoses of CV disease and diabetes mellitus were confirmed from the medical records.

Participants fasted overnight, and venous blood samples were taken by trained staff and immediately sent to the accredited Laboratory of Clinical Chemistry, Västmanland County Hospital, Västerås. The estimated glomerular filtration rate (eGFR) was calculated from creatinine levels standardized by isotope dilution mass spectrometry (SYNCHRON LX or UniCel DxC instruments; Beckman Coulter, Inc., Brea, CA, USA) using the Chronic Kidney Disease Epidemiology Collaboration (CKD-EPI) formula (28).

Glycated hemoglobin (HbA1c) mono was determined using a TOSOH Glycohemoglobin Analyzer G7 (Tosoh, Japan) and calibrated against the Swedish Mono-S method. The HbA1c was calculated from HbA1c mono using the formula: 10.45 × (HbA1c mono level) – 10.62 (International Federation of Clinical Chemistry and Laboratory Medicine, IFCC standard). The treatment target level for diabetics (≤53 mmol/mol) was based on the European Society of Cardiology (ESC) guidelines from 2003 (29).

The serum total cholesterol (TC) concentration was determined using a UniCel DxC 800 or SYNCHRON LX20 Analyzer (Beckman Coulter, Inc.). Levels of low-density lipoprotein cholesterol (LDL-cholesterol) were calculated from those of TC, high-density lipoprotein cholesterol (HDL-cholesterol), and triglycerides using the Friedewald equation: (LDL-cholesterol = TC – HDL-cholesterol – Triglyceride levels × 0·45). The treatment target levels of LDL-cholesterol are based on the ESC guidelines from 2003 (<2.5 mmol/L) and 2019 (<1.4 mmol/L) (29, 30).

Based on a standard 12-lead surface electrocardiography (ECG), left ventricular (LV) hypertrophy was defined as the Sokolow–Lyon voltage >35 mV or Cornell voltage >28 mV in men and >20 mV in women.

### Ankle blood pressure and carotid ultrasound

Blood pressure in both arms and ankles was measured in all included participants in a supine position after at least 5 min rest. The ankle BP was measured in the bilateral dorsalis pedis and posterior tibial arteries using an inflatable leg-cuff, an aneroid sphygmomanometer, and a handheld Doppler instrument with a 5-MHz probe. The ABI was calculated by dividing the highest ankle pressure by the highest BP of both arms. An abnormal ABI was defined as ≤0.90 or ≥1.40 in either leg.

Carotid artery ultrasonography has been described in detail (26). Briefly, grading of ICA lesions into normal artery, plaque without flow disturbance, mild/moderate/severe stenosis, or occlusion was based on gray-scale images, color flow Doppler scans, and spectral Doppler blood flow velocities.

### Clinical and ambulatory BPs

Clinical BP was measured manually by trained technicians and was obtained from the non-dominant arm or from the other arm if the systolic BP was >10 mmHg higher. The BP was measured from participants in the supine position after at least 5 min rest and was rounded up to the nearest 2 mmHg. Using the arm from which clinical BP was obtained, the ABPM 04 instrument (Meditech Ltd., Budapest, Hungary) was applied for 24-h ABPM with readings taken every 20 min (31). Three different cuff-sizes were available and selected depending on the size of the patient’s upper arm. Day- and night-time periods were assessed from the time of awakening and sleeping entered by the patient in a diary card.

The clinical BP was defined as poorly controlled if systolic BP was ≥140 mmHg or diastolic BP was ≥90 mmHg. The corresponding definition for ambulatory BP was a 24-h ambulatory systolic BP of ≥130 mmHg or diastolic BP ≥80 mmHg (10, 32). White coat hypertension was defined as a poorly controlled clinical BP combined with a well-controlled ambulatory BP, whereas masked hypertension was defined as the reverse condition, that is, a well-controlled clinical BP in combination with a poorly controlled ambulatory BP (20).

### Statistics

Data are presented as mean ± standard deviation or frequency and (percentage). To investigate potential predictors of a poorly controlled ambulatory BP, we used logistic regression analysis. In a multivariable model, we included available variables that have been proposed or established in previous studies: age, sex, body mass index (BMI), smoking habit, educational level, living alone, physical activity, diabetes, eGFR, previous myocardial infarction (MI), stroke, heart failure, abnormal ABI, ICA stenosis, number of antihypertensive medications, LV hypertrophy, and clinical systolic and diastolic BP (14–18). Statistical analyses were performed using R 3.5.3 (R Foundation for Statistical Computing, 2019, Vienna, Austria; http://www.r-project.org). Two-sided *P* values <0.05 were considered to be statistically significant

## Results

### Baseline characteristics

Nearly a third of the patients (28%) were smokers (Table 1). Among patients with a poorly controlled ambulatory BP, diabetes mellitus was more frequent (29%) compared with the those with well-controlled ambulatory BP (19%). ICA stenosis and LV hypertrophy were more prevalent among patients with poorly controlled ambulatory BP. Most of the patients (53%) had an LDL-cholesterol level of ≥2.5 mmol/L, and almost all reported a level of ≥1.4 mmol/L. Of the 97 patients with diabetes mellitus, 53 patients (55%) had an HbA1c level of >53 mmol/mol, whereas this was seen only in six (2%) of the 305 patients without documented diabetes.

**Table 1:** Characteristics of patients with peripheral arterial disease, overall and stratified by well-controlled (<130/80 mmHg) and poorly controlled (≥130/80 mmHg) ambulatory 24-h blood pressure (BP).

	All patients; *n* = 402	Well-controlled ambulatory (Amb) BP; *n* = 194	Poorly controlled Amb BP; *n* = 208
Age (years)	69.9 ± 7.1	69.6 ± 7.2	70.1 ± 7.1
Male (sex)	240 (59.7%)	118 (60.8%)	122 (58.7%)
Body mass index (kg/m^2^)	27.0 ± 4.2	26.3 ± 3.9	27.7 ± 4.3
Smoking	112 (28.1%)	55 (28.9%)	57 (27.4%)
Low education level	229 (57.0%)	116 (59.8%)	113 (54.3%)
Living alone	105 (26.2%)	58 (29.9%)	47 (22.8%)
Physically inactive	108 (26.9%)	51 (26.3%)	57 (27.4%)
Diabetes mellitus	97 (24.1%)	37 (19.1%)	60 (28.8%)
Abnormal ankle-brachial index	225 (56.0%)	109 (56.2%)	116 (55.8%)
Internal carotid artery stenosis	303 (75.4%)	139 (71.6%)	164 (78.8%)
Previous myocardial infarction	78 (19.4%)	39 (20.1%)	39 (18.8%)
Previous stroke	42 (10.4%)	22 (11.3%)	20 (9.6%)
Heart failure	29 (7.2%)	19 (9.8%)	10 (4.8%)
Total cholesterol (mmol/L)	4.56 ± 1.16	4.47 ± 1.11	4.65 ± 1.20
Low-density lipoprotein (LDL) cholesterol (mmol/L)	2.68 ± 0.94	2.63 ± 0.90	2.73 ± 0.98
LDL cholesterol ≥2.5 mmol/L	210 (53.3%)	100 (52.4%)	110 (54.2%)
LDL cholesterol ≥1.4 mmol/L	385 (97.7%)	185 (96.9%)	200 (98.5%)
Estimated glomerular filtration rate (mL/min/1.73 m^2^)	73.9 ± 17.5	73.8 ± 16.4	73.9 ± 18.4
Glycated hemoglobin (mmol/mol)	42.3 ± 10.3	41.0 ± 8.3	43.4 ± 11.8
Left ventricular hypertrophy	66 (16.7%)	23 (12.0%)	43 (21.0%)
Angiotensin-converting enzyme inhibitor/Angiotensin receptor blocker	230 (57.2%)	108 (55.7%)	122 (58.7%)
Betablockers	200 (49.8%)	103 (53.1%)	97 (46.6%)
Diuretics	96 (23.9%)	50 (25.8%)	46 (22.1%)
Calcium inhibitor	145 (36.1%)	64 (33.0%)	81 (38.9%)
Statins	329 (81.8%)	161 (83.0%)	168 (80.8%)
Blood pressure			
Clinical systolic blood pressure (SBP) (mmHg)	153 ± 21	143 ± 17.7	162 ± 19.7
Clinical diastolic blood pressure (DBP) (mmHg)	76.7 ± 9.69	74.5 ± 8.8	78.8 ± 10
Amb 24-h SBP (mmHg)	131 ± 13.9	120 ± 7.09	141 ± 10.3
Amb 24-h DBP (mmHg)	68 ± 8.49	64.5 ± 6.93	71.2 ± 8.55

Values are presented as mean ± SD or frequency (percentage). There were missing values in the following categories: smoking (*n =* 4), living alone (*n =* 2), heart failure (*n =* 1), total cholesterol (*n =* 2), LDL cholesterol (*n =* 8), eGFR (*n =* 1), and LV hypertrophy (*n =* 6).

### Clinical and ambulatory BPs

**Figure 1:**
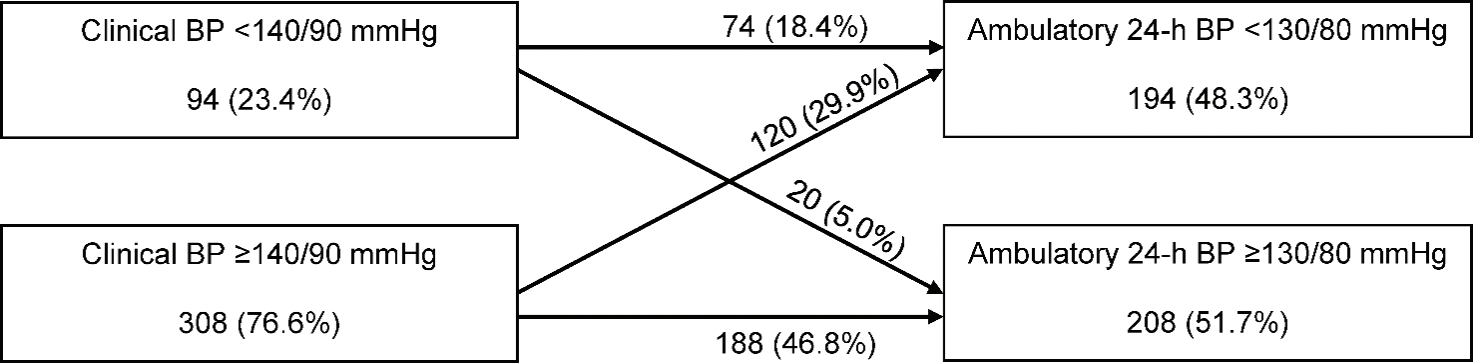
Flowchart showing the prevalence of well and poorly controlled levels of clinical and ambulatory 24-h blood pressure (BP) in 402 outpatients with peripheral arterial disease.

Most of our patients with PAD had poorly controlled BPs, especially clinical BP (76.6%), but also ambulatory BP (51.7%) (Figure 1). A clinical systolic BP of ≥140 mmHg was found in 307 (76.4%) patients, and a clinical diastolic BP of ≥90 mmHg was observed in 58 (14.4%) patients. If a poorly controlled clinical BP was re-defined as systolic BP >140 mmHg or diastolic BP >90 mmHg, the prevalence rate was found to be 70.4% (*n* = 283). Results of ABPM revealed that 120 (29.9%) patients exhibited white coat hypertension and 20 (5.0%) patients showed masked hypertension. An ambulatory 24-h systolic BP of ≥130 mmHg was detected in 207 (51.5%) patients and a 24-h diastolic BP of ≥80 mmHg observed in 40 (10.0%).

**Figure 2:**
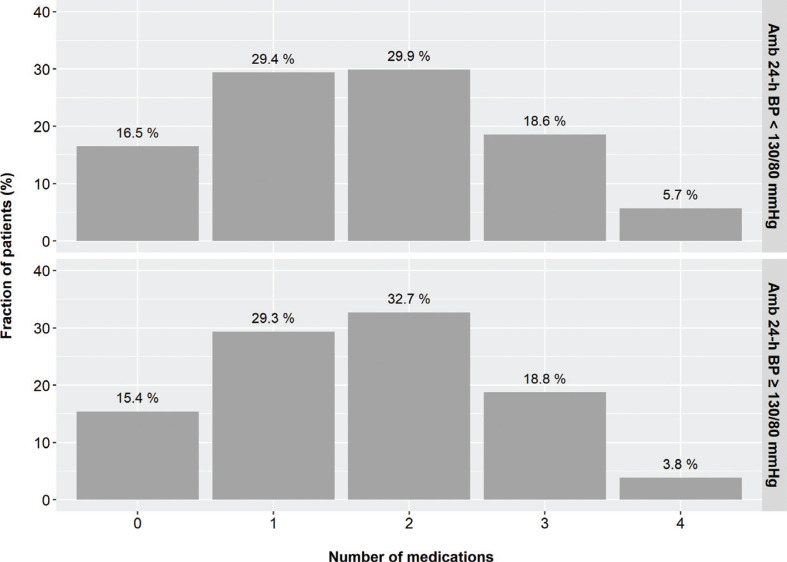
The distribution of the number of prescribed antihypertensive medications according to ambulatory blood pressure levels.

Antihypertensive medications were prescribed in 84% of patients. The number of drugs was remarkably similar in patients with well-controlled and poorly controlled ambulatory BP (Figure 2). More than 40% of the patients were taking only 0 – 1 medication, while less than 25% of patients were using three or more agents.

### Predictors of poorly controlled ambulatory BP

**Figure 3:**
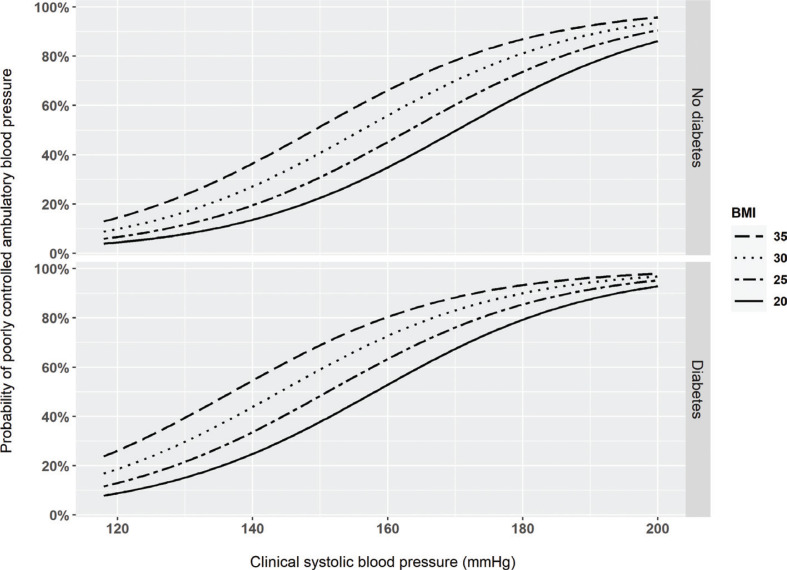
Probability of poorly controlled ambulatory 24-h blood pressure (i.e. ≥130/80 mmHg) according to the clinical systolic blood pressure, body mass index (BMI) in kg/m^2^, and occurrence of diabetes mellitus. The probabilities are adjusted for the variables shown in Table 2.

In a logistic regression analysis, higher clinical systolic BP, higher BMI, fewer antihypertensive medications, and diabetes mellitus were independent predictors of a poorly controlled ambulatory BP independently (Table 2). Clinical diastolic BP was not associated with poorly controlled ambulatory BP. In Figure 3, the probability of poorly controlled ambulatory BP is illustrated depending on the diabetic status and levels of clinical systolic BP and of BMI after adjustment for the other variables listed in Table 2. Patients with diabetes mellitus had similar clinical systolic BP profiles compared with those without diabetes (154 ± 20 vs. 153 ± 21 mmHg; *P* = 0.604) but a higher ambulatory 24-h systolic BP (135 ± 15 vs. 129 ± 13 mmHg; *P* = 0.002).

**Table 2:** Clinical characteristics independently associated with a poorly controlled 24-h ambulatory blood pressure (i.e. ≥130/80 mmHg) in patients with peripheral arterial disease.

	Odds ratio (95% CI)	*p*
Age (for every 10-year increase)	0.79 (0.52–1.21)	0.281
Male (sex)	1.00 (0.60–1.66)	0.998
Body mass index	1.09 (1.02–1.17)	0.009
Smoking	1.23 (0.68–2.23)	0.486
Low education	0.79 (0.48–1.31)	0.370
Living alone	0.81 (0.47–1.42)	0.464
Physically inactive	1.04 (0.60–1.80)	0.900
Diabetes	2.03 (1.10–3.75)	0.024
Estimated glomerular filtration rate (for every 10-unit increase)	0.94 (0.80–1.10)	0.412
Previous myocardial infarction	1.18 (0.60–2.33)	0.632
Stroke	0.79 (0.35–1.78)	0.563
Heart failure	0.35 (0.11–1.11)	0.074
Abnormal ankle–brachial index	1.11 (0.64–1.93)	0.708
Internal carotid artery stenosis	1.44 (0.77–2.69)	0.251
Number of medications	0.67 (0.52–0.86)	0.001
Left ventricular hypertrophy	1.87 (0.95–3.68)	0.071
Clinical systolic blood pressure (for every 10-unit increase)	1.81 (1.52–2.15)	<0.001
Clinical diastolic blood pressure (for every 10-unit increase)	1.00 (0.74–1.35)	0.998

Values are odds ratios and 95% confidence intervals (CI) for every one unit increase in continuous independent variables, unless stated otherwise. Analysis is based on 388 patients in a multivariable logistic regression model.

## Discussion

We found that both clinical and ambulatory systolic BPs were poorly controlled in our outpatients with PAD, and that many of these patients appeared to have undergone suboptimal treatment with few antihypertensive medications. Patients with diabetes had an increased risk of poorly controlled ambulatory BP. Moreover, a high proportion of the patients were still smokers and with hyperlipidemia, further emphasizing that preventive care against CV risk factors could be improved in such patients. Among the patients with poorly controlled clinical BP, one-third had white coat hypertension, whereas only 5% of the patients exhibited masked hypertension. BMI, diabetes mellitus, number of medications, and clinical systolic BP were independent predictors of a poorly controlled ambulatory BP.

The results of this study suggest that a substantial proportion of outpatients with PAD have undertreated systolic hypertension. In contrast, diastolic BPs were better controlled, which is in accordance with previous findings in patients with PAD and likely derives from the stiffness of arteries caused by atherosclerosis (32).

Elevated BP is the most important risk factor for death and disability worldwide, accounting for almost 10 million deaths in 2015 (13). Only a few previous studies have investigated ambulatory BP levels in patients with PAD. Skoglund et al. evaluated clinical and ambulatory BP profiles in 98 male patients with lower extremity arterial disease (33). Compared with their findings, our patients had similar mean clinical systolic and diastolic BPs (153/77 vs. 151/79 mmHg), whereas the mean ambulatory 24-h BPs were lower in our population (131/68 vs. 142/78 mmHg). This might have been because of a higher rate of antihypertensive medications (84% vs. 70%) in our population.

Data on optimal BP treatment target levels in patients with PAD are conflicting. Possibly, lowering BP too far in these patients might reduce perfusion in lower limbs and increase the risk of PAD-related events. In a reanalysis of data from the Antihypertensive and Lipid-Lowering Treatment to Prevent Heart Attack Trial (ALLHAT), the association of clinical BPs with incident PAD events (hospitalization, procedures, medication, or death related to PAD) was evaluated. The authors found a U-shaped association of systolic BP with PAD events. Higher rates of lower extremity PAD events were observed with high (>160 mmHg) as well as with low (<120 mmHg) systolic BP and with low diastolic BP (<70 mmHg) (34). In contrast, a meta-analysis of small prospective studies suggested that antihypertensive treatment was not associated with worsening symptoms or outcomes in PAD, and there was a trend toward improvement in leg ischemia (35). Although optimal BP target levels specific for PAD are unclear, the evidence so far has not been strong enough to modify guidelines for patients with PAD (34). The suboptimal treatment of hypertension remains worrying, because patients with PAD are at high risk of CV-related mortality (9, 36) but are less intensively treated and have a higher CV mortality than those with MI (37).

A combination of drugs is recommended as first-line therapy against hypertension for most patients according to the latest guidelines (10). In a recent Swedish nationwide registry study of long-term prophylactic treatment patterns in PAD, 60% of the patients had any antihypertensive treatment (8), which is considerably lower than the 84% in our study population. Nonetheless, there was a high prevalence of poorly controlled hypertension in our patient population, and more than 40% of patients were using only one or no BP-lowering agents.

In the general population, white coat hypertension can be found in up to 30–40% of subjects with an elevated in-clinic BP level, and masked hypertension can be observed in around 15% of patients with a normal in-clinic BP level (10). Ambulatory BP in our patients revealed fewer cases with masked hypertension (5%) and a similar proportion with white coat hypertension (30%). These findings suggest that ABPM is of minor importance in detecting masked hypertension in patients with PAD. Instead, ABPM may be more useful in detecting white coat hypertension when attempts to reduce clinical BPs fail.

The clinical features that predicted a high ambulatory BP were not surprising. The prevalence of hypertension is known to be increased in individuals with other CV risk factors, such as obesity and diabetes (30, 31). That high clinical BP increases the risk of poorly controlled ambulatory BP and that the use of more hypertensive medications reduces BP, was also expected.

In parallel with poorly controlled BP profiles, it is obvious that the control of other important CV risk factors was weak in our population. Nearly 30% of our patients were smokers, which is markedly more common than in the general population, where the prevalence of smoking in Sweden has decreased from 14% to 7% between 2006 and 2018 according to the Public Health Agency of Sweden. Smoking is a particularly strong risk factor for PAD (1), and smoking duration seems to be a risk factor for women after only 10 years of smoking (36). Smoking cessation is highly beneficial and should be prioritized to reduce CV events and mortality rates (2).

The latest European guidelines recommend lipid-lowering treatments in all patients with PAD, including a maximum tolerated dose of statins, plus ezetimibe or in combination with a proprotein convertase subtilisin/kexin type 9 (PCSK9) inhibitor if needed. The use of statins and addition of a PCSK9 inhibitor to further lower the LDL-cholesterol level seem to provide a favorable effect on lower limb prognosis, in addition to further reduction of CV events (38, 39). A nationwide Swedish study found that 74% of patients with MI used statins, but only 53% of patients with PAD did so (37). Among our patients, more than 80% were using statins; however, the majority of these individuals still had excessive levels of LDL-cholesterol (Table 2).

Diabetes mellitus is strongly associated with an elevated risk of PAD and with worse outcomes (1, 10). Here, outpatients with PAD and concomitant diabetes had an increased risk of poorly controlled ambulatory BP.

That many of our outpatients with PAD were still smokers and with LDL-cholesterol levels of ≥2.5 mmol/L, and that many with diabetes had HbA1c levels >53 mmol/mol, in addition to poorly controlled hypertension, suggests the need for improved preventive care in such individuals.

This research study was limited to consecutive outpatients of European origin who were found to have lower extremity and/or carotid artery disease in a visit to a vascular ultrasound laboratory. The invited patients who declined to join the study (*n* = 162) did not differ in age (*P* = 0.68) or sex (*P* = 0.93) compared with the participants. However, if more burdened with disease, these dropouts may have been a source of bias. The participants who declined ABPM (*n* = 35) or were excluded due to few ABPM readings (*n* = 15) did not differ significantly from the included participants regarding age, sex, BMI, smoking, diabetes, previous myocardial infarction, or stroke (all *p*-values >0.095). Data on medication were based on self-reported information, which might be a cause of informational bias. However, good agreement between patient interview and computerized pharmacy records has been found in the elderly population (40). The clinical BPs were measured on one occasion only. Furthermore, we did not have any information regarding previous efforts to intensify antihypertensive treatments in these patients.

In our outpatients with PAD, diagnosed in a vascular ultrasound laboratory, poorly controlled clinical and ambulatory systolic BP profiles were common. Those receiving a higher number of antihypertensive agents had a better BP control, thus suggesting room for improvement. In addition, we found a suboptimal control of other important CV risk factors in these patients, including smoking, HbA1c levels in those with diabetes mellitus, and LDL-cholesterol levels. This study will help in motivating intensified preventive efforts against CV complications in outpatients with PAD.
